# Supplementary material for: Content-rich biological network constructed by mining PubMed abstracts
Source: BMC Bioinformatics. 2004 Oct 8;5:147. doi: 10.1186/1471-2105-5-147 (PMC528731; doi:10.1186/1471-2105-5-147)
Supplement: Additional File 2 — The original results of the above study (non-essential files are deleted to keep the file size under the limit set by BMC bioinformatics). [file 1471-2105-5-147-S2.bz2 › chilibotAdditionalFile2/dip05/229ID7961947E747/html/HSPD1_MPST.html]

 


 **HSPD1** and **MPST** 
  
Found 2 abstracts in PubMed, retrieved 2.  
 

 What does Google say? 
 PDF only 
| .edu only 

---

- Clin Exp Immunol, 1997   **Polyclonal T cell elimination by prolonged immunostimulation in an experimental model.** .
  An experimental model of immunological deficiency obtained by treating mice for 6 months with serum of human blood drawn from different healthy individuals has been studied.
  The results show that an alteration of a circulating lymphocyte population with alterations of the ratio CD4 CD8 appeared in mice stimulated for a long period with immunogens.
  Mice treated for 2 4 months showed an increase in B lymphocytes and a decrease in the total number of T lymphocytes, with a decrease in CD4 lymphocytes and an increase in CD8 lymphocytes.
  After 4 months, the CD8 lymphocyte population started to decrease, with a ratio of CD4 CD8 reaching almost 1.
  In animals treated for 2 3 months, the mean survival time MST **[MPST]** following experimental infection with Salmonella typhimurium presented a decrease to 5 days, and after 5 6 months of treatment presented a decrease to 3 2.5 days.
  The bacteraemia was modified in comparison with controls.
  Prolonged exposure to antigens also induced lymphocyte apoptosis cells of animals treated for 4 6 months presented increased levels of apoptosis with a percentage that reached 30 35%.
  A semiquantitative evaluation of the level of heat shock protein hsp in splenic lymphocytes showed an increase in the presence of hsp60 **[HSPD1]** and hsp70 in the first 3 months of treatment, which then remained constant for up to 6 months.

  - J Biol Chem, 1991   **Chaperonins facilitate the in vitro folding of monomeric mitochondrial rhodanese.** .
    In vitro refolding of the monomeric mitochondrial enzyme, rhodanese thiosulfate sulfurtransferase **[MPST]**.
    EC 2.8.1.1 is facilitated by molecular chaperonins.
    The four components two proteins from Escherichia coli, chaperonin **[HSPD1]** 60 groEL **[HSPD1]** and chaperonin **[HSPD1]** 10 groES , MgATP, and K , are necessary for the in vitro folding of rhodanese.
    These were previously shown to be necessary for the in vitro folding of ribulose bisphosphate carboxylase at temperatures in excess of 25 degrees C Viitanen, P.
    V.
    , Lubben, T.
    H.
    , Reed, J.
    , Goloubinoff, P.
    , O Keefe, D.
    P.
    , and Lorimer, G.
    H.
    1990 Biochemistry 2 665 5671 .
    The labile folding intermediate, rhodanese I, which rapidly aggregates at 37 degrees C in the absence of the chaperonins, can be stabilized by forming a binary complex with chaperonin **[HSPD1]** 60.
    The discharge of the binary chaperonin **[HSPD1]** 60 rhodanese I complex, results in the formation of active rhodanese, and requires the presence of chaperonin **[HSPD1]** 10.
    Optimal refolding is associated with a K dependent hydrolysis of ATP.
    At lower protein concentrations and 25 degrees C, where aggregation is reduced, a fraction of the rhodanese refolds to an active form in the absence of the chaperonins.
    This spontaneous refolding can be arrested by chaperonin **[HSPD1]** 60.
    There is some refolding approximately equal to 20% when ATP is replaced by nonhydrolyzable analogs, but there is no refolding in the presence of ADP or AMP.
    ATP analogs may interfere with the interaction of rhodanese I with the chaperonins.
    Nondenaturing detergents facilitate rhodanese refolding by interacting with exposed hydrophobic surfaces of folding intermediates and thereby prevent aggregation Tandon, S.
    , and Horowitz, P.
    1986 J.
    Biol.
    Chem.
    26 5615 15618 .
    The chaperonin **[HSPD1]** proteins appear to play a similar role in as much as they can replace the detergents.
    Consistent with this view, chaperonin **[HSPD1]** 60, but not chaperonin **[HSPD1]** 10, binds 2 3 molecules of the hydrophobic fluorescent reporter, bi 4 anilino naphthalene S,5 disulfonic acid, indicating the presence of hydrophobic surfaces on chaperonin **[HSPD1]** 60.
    The number of bound probe molecules is reduced to 1 2 molecules when chaperonin **[HSPD1]** 10 and MgATP are added.
    The results support a model in which chaperonins facilitate folding, at least in part, by interacting with partly folded intermediates, thus preventing the interactions of hydrophobic surfaces that lead to aggregation.
